# Open questions on the transfer of chirality

**DOI:** 10.1038/s42004-021-00614-y

**Published:** 2021-12-09

**Authors:** Jolene P. Reid

**Affiliations:** Department of Chemistry, University of British Columbia, 2036 Main Mall, Vancouver, British Columbia V6T 1Z1 Canada

**Keywords:** Stereochemistry, Reaction mechanisms, Asymmetric catalysis

## Abstract

The transfer of chiral information from optically pure reaction components to products can generate enantiomerically-enriched molecules, but the control of stereochemistry often proves challenging. Here, the author highlights how our fundamental understanding of stereocontrol has evolved and discusses possible approaches for the rational development of enantioselective catalysts.

Chiral transfer usually arises in a chemical reaction when stereochemical information is transmitted from one reaction component (substrate, reagent, catalyst, and solvent) to the product. Such information is most effectively relayed through highly ordered transition states (TSs) wherein, molecular interactions between chiral and achiral molecules lead to one pathway being strongly favored over other possibilities (Fig. 1a)^1^. Historically, most approaches to chiral molecule construction centered on substrate or reagent control. In this mechanistic scenario, one chiral molecule of starting material produces a single molecule of product with high levels of stereoselectivity. Such processes have been studied extensively both as a fundamental phenomenon and for synthetic utility. Importantly, the lack of substrate effects on the product outcome has enabled such systems to be rendered impressively robust and the stereochemistry decidedly certain. Thus, many of these procedures are still routinely employed for complex molecule synthesis.

**Fig. 1:**
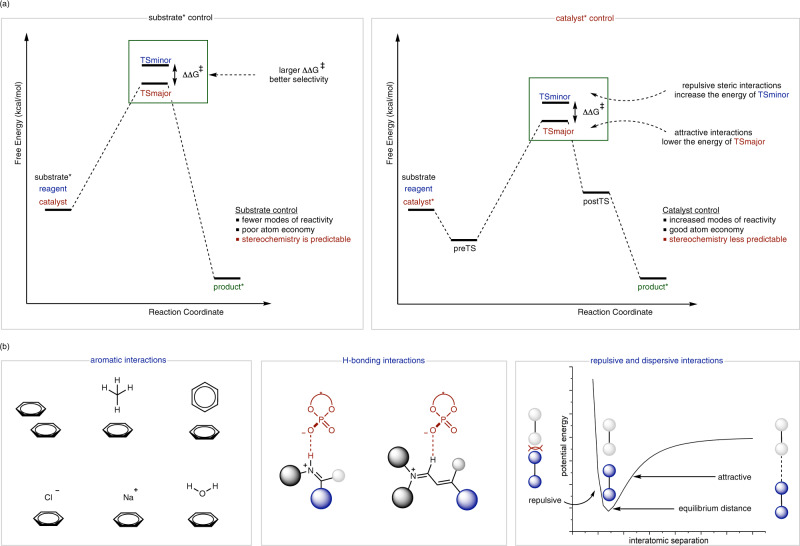
The fundamentals of chiral transfer in organic synthesis. **a** Comparing the two key approaches for chiral molecule generation where TS_major_ and TS_minor_ lead to the favored and competing enantiomers. ΔΔG^‡^ is the energy difference between the TSs, the reaction coordinate depicts the progression of the reaction with preTS and postTS indicating stationary points that connect the starting and product structure to the TS. **b** Key noncovalent interactions (NCIs) that occur between chiral and achiral reaction components to determine the energies and geometries of TSs. The plot on the right is the Lennard-Jones that describes the potential energy of interaction between two non-bonding atoms based on their separation distance.

The invention of catalytic methods to facilitate bond constructions has expanded the reach of chiral transfer to new reactions with increased efficiency. The complicated conditions and complex catalyst structures of modern reactions make it difficult to anticipate how the reaction components organize at the TS to impart selectivity. This leads to the questions: (1) what fundamental interactions guide the transfer of chirality, (2) how can these be identified and understood, and (3) is it possible to harness this understanding for effective catalyst design? This text provides a brief overview of how the research community is currently confronting these questions and future directions in the field.

## What are the molecular interactions determining the transfer of chirality?

The geometries and energies of TSs that lead to competing products are defined by noncovalent interactions (NCIs) which are expressed in several different forms (CH−π, π−π, electrostatic, etc.) and are energetically weak (the energy increments associated with these interactions are normally modest ~2–3 kcal/mol). Therefore, the characterization of many NCIs is generally difficult, yet necessary for understanding the factors required for effective stereoinduction (Fig. 1b)^2^. The diversity of NCIs is extensive but the underlying physical origins of how they operate are fundamentally similar. Often these involve an interaction between two systems separated by space where the contact strength is attenuated by the types of atoms/molecular orbitals involved, distance, and direction. Although, such similarities can make it difficult to recognize the specific interactions at the TS as many NCIs can account for the observed outcome. Analyzing how the enantiomeric excess (ee) changes as a function of catalyst and/or substrate structure can offer some clues into the types of NCIs that may be present at the relevant TSs^3^. Such experiments provide a firm basis for computationally exploring the precise interactions determining reaction outcomes. In other words, experimental data is incredibly useful to generate mechanistic hypotheses which can be further refined by calculations. This is important for exhaustively considering all possibilities as practitioners are much less likely to locate TSs that they have not purposefully sought to identify. A relevant example was recently disclosed by our group in the context of asymmetric counteranion-directed catalysis involving chiral phosphates and iminium intermediates^4^. The theoretical investigation led to the characterization of an NCI that was not previously investigated in asymmetric catalysis and revealed a new explanation for the enantioselectivity outcome. More specifically, the TS that leads to the major enantiomer was found to be stabilized by a CH···O hydrogen bonding interaction between the iminium intermediate and the chiral phosphate, worth approximately 3.9 kcal/mol. Given the strength of the NCI and the importance of iminiums as intermediates in asymmetric catalysis, it is reasonable to suggest that this interaction will be considered important in many other reaction systems. A subsequently emerging question is whether additional NCIs have not yet been recognized as meaningful stereocontrolling elements?

In this context, the accuracy of the computational approach is also of significance. Only relatively recently have theoretical methods been developed that are able to model attractive NCIs accurately^5^. Because several selectivity models were derived from calculations prior to the introduction of dispersion containing functionals, reactions are continuously being computationally re-evaluated allowing our mechanistic understanding of the reasons for selectivity to be refined^6^. The Schreiner group recently demonstrated this issue in the context of the well-known Corey–Bakshi–Shibata (CBS) reduction^7^. While many classical applications of this reaction for the generation of chiral alcohols from ketones suggest simple steric control, calculations with modern functionals show that the reaction outcome is not only due to such destabilizing effects. Indeed, besides repulsive interactions between substrate and catalyst, the computations show that enantioselectivity trends can only be fully explained by also taking into account stabilizing contacts. Therefore, it is probable that many other important processes remain only partially understood, hindering the application and development of such catalytic systems.

## What approaches are available for catalyst design?

Computational methods can also be used to design catalysts for enantioselective transformations^8^. Several notable examples are known and the greatest successes have involved modifying catalysts that exhibit some desirable properties and making modifications to the structure to logically suggest new systems with the desired function. As an oft-cited organocatalytic example, (*S*)-proline is well known to catalyze Mannich reactions with high levels of enantio- and diastereoselectivity. Computations indicated that selectivity arises from the *s-trans* enamine geometry and the approach of the reactant from the front. If the catalyst was re-designed to promote the reaction through the *s-cis* geometry a highly selective *anti*-Mannich reaction could be possible. Consequently, a modified proline catalyst bearing a methyl substituent that had not been previously prepared was evaluated computationally and subsequently verified by experiment^9^.

The computational assessment of several catalyst complexes can be a computer-intensive process for establishing which properties determine the enantioselectivity and proposing new catalysts that may lead to an improvement. An alternative method is to discover connections between catalyst descriptors derived from simple ground-state structures and selectivity data^10^. Such relationships can rapidly uncover the important structural features that are necessary for efficient stereoinduction allowing rational modifications to be made. Implementation of this approach for catalyst development generally requires only moderate amounts of data (10 s of points) but correlations can be difficult to construct if stereoinduction does not follow reasonable catalyst features. Despite this, several reports have demonstrated the use of this tactic for the optimization of higher selectivity chiral phosphoric acids^11,12^ and diamine ligands^13^.

However, the design of catalysts for facilitating reactions outside of an existing framework is much more difficult. Building such systems from the ground up would require conceiving a target transformation, and then deploying computations to predict a catalyst that would facilitate that reaction. In this regard, perhaps, the identification of feasible mechanisms that lead to both desired and competing products is the most challenging task. Several research groups have developed new computational approaches that enable the potential energy surface to be explored in a less biased manner^14,15^. Ultimately, this allows practitioners to identify and consider additional mechanistic possibilities. Although, such methods are generally too slow to be competitive with experiments and application to complex catalytic systems have not yet been fully evaluated, further research in this area is necessary. Could it be that there will be a significant intersection of advances in mechanistic understanding and reaction informatics that will allow progress to be made in this arena?

## Is a certain catalyst best suited to a specific function?

It is well-recognized that different catalyst structures are better suited for certain transformations, but linking particular catalysts to distinct reactions is rather difficult and only a few investigations of this type have been performed^16^. However, it has long been known that many catalysts can effectively transfer chirality to a diverse range of products^17^. Nonetheless, detailed insight into how such “privileged” catalysts operate so generally was only recently disclosed. By using computations and physical organic correlations our group showed that the principles underlying effective asymmetric catalysis involving chiral phosphoric acids and their conjugate bases were fundamentally the same^4^. We anticipated that this was likely a general phenomenon in asymmetric catalysis, whereby various mechanistically unrelated transformations would be found to perform similarly when the key catalyst and substrate components are conserved. Shortly after our report, Jacobsen and co-workers demonstrated that the findings we deduced from our study also extended to hydrogen bond donor catalysis^18^. Therefore, a pressing and relevant question is if it is possible in principle to design general catalysts? In considering this question it is imperative to survey what structural elements constitute the broad applicability of certain catalysts. An intriguing example of how catalyst flexibility can be beneficial for imparting selectivity across a wide substrate scope was recently reported by the Miller, Sigman, and Toste groups^19^. The transformation developed was an atropisomer-selective cyclodehydration of *o*-substituted aniline derivatives to afford benzimidazoles. During the reaction optimization process, two catalysts classes were found to be effective: (1) the rigid *C*_2_-symmetric BINOL-derived chiral phosphoric acids and (2) the considerably less explored, flexible, peptide-based phosphoric acids. After independently optimizing each catalyst system, a set of 20 structurally diverse substrates was evaluated using the lead catalyst from each class. Surprisingly, both catalyst types performed well for the vast majority of substrates (>90% ee), however, the peptide-based phosphoric acids appeared to promote high atroposelectivity for a wider assortment of substrates. To probe these differences in substrate performance, two structure-selectivity correlations were built for each separate catalyst class using DFT-derived parameters that describe the substrate features and a forward stepwise multivariate linear regression algorithm. As revealed by the overlapping terms between the two statistical models, the vast majority of the interactions between catalyst and substrate were similar. However, a clear difference in the models is the result of steric effects in which BINOL-derived chiral phosphoric acids lead to lower enantioselectivities with substrates incorporating large, bulky substituents. In contrast, the peptide appears to function through an alternative mode of enantioinduction, where conformational adaptation presumably limits repulsive interactions explaining why this catalyst is less sensitive to the steric demands on the substrate. The question is how general can an enantioselective catalyst be?

## Outlook

The successful transfer of chirality from catalyst to product for any target transformation is an exciting prospect. Evidently, the community is still far from achieving this ambitious goal, but as noted throughout, significant progress has been made in this direction. One can anticipate further computational and synthetic studies will focus on exploring the precise molecular interactions governing efficient stereoinduction from which new understandings and better catalyst systems will emerge. The extension of these approaches to large, *C*_1_-symmetric and flexible systems, for which multiple conformations actively participate in chemical reactions, is a particularly daunting task. But incorporating such non-intuitive structural features into catalyst design may offer new systems with unexpected generality. Ultimately, further progress in this area will be achieved by combining our current understanding with new technologies (theory, hardware, artificial intelligence, etc.).
